# Pretreatment L3 Skeletal Muscle Index Is Associated with Severe Oral Intake Impairment and a Longer Treatment-to-Discharge Interval in Patients with Head and Neck Squamous Cell Carcinoma Receiving Definitive Chemoradiotherapy

**DOI:** 10.3390/cancers18162677

**Published:** 2026-08-18

**Authors:** Yasuhiro Fukushima, Tomohiro Shimizu, Tomotaka Kakubari, Soma Kumasaka, Daisuke Ozaki, Yusuke Sato, Tomokazu Takeuchi, Masaomi Motegi, Kazuaki Chikamatsu, Yoshito Tsushima

**Affiliations:** 1 Department of Radiological Sciences, Faculty of Medical Science and Technology, Gunma Paz University, Takasaki 370-0006, Japan; y-fukushima@paz.ac.jp; 2 Department of Otolaryngology–Head and Neck Surgery, Gunma University Graduate School of Medicine, Maebashi 371-8511, Japankakutomo95@gmail.com (T.K.); tikamatu@gunma-u.ac.jp (K.C.); 3 Department of Applied Medical Imaging, Gunma University Graduate School of Medicine, Maebashi 371-8511, Japan; kumasaka88@gunma-u.ac.jp; 4 Department of Radiology, Gunma University Hospital, Maebashi 371-8511, Japan; dozaki@gunma-u.ac.jp (D.O.);; 5 Center for Advanced Hearing Implants and Hearing Aids, Gunma University Hospital, Maebashi 371-8511, Japan; 6 Department of Diagnostic Radiology and Nuclear Medicine, Gunma University Graduate School of Medicine, Maebashi 371-8511, Japan

**Keywords:** head and neck squamous cell carcinoma, sarcopenia, skeletal muscle index, body composition, chemoradiotherapy, dysphagia, deep learning, computed tomography

## Abstract

**Background/Objectives**: Pretreatment sarcopenia predicts adverse outcomes in head and neck squamous cell carcinoma (HNSCC). The conventional L3 skeletal muscle index (SMI) requires abdominal imaging, whereas the predictive value of the more accessible C3 muscle index for acute treatment-related complications remains uncertain. We compared pretreatment L3 SMI and the C3 index for identifying patients at risk of severe oral intake impairment, radiation interruption, and a longer treatment-to-discharge interval during definitive chemoradiotherapy. **Methods**: This retrospective single-center study included 55 patients with HNSCC treated with 80 mg/m^2^ cisplatin every three weeks and 66 Gy intensity-modulated radiotherapy. Pretreatment computed tomography (CT) was analyzed using deep-learning-based automated segmentation. Firth-penalized logistic and multivariable linear regression were adjusted for age, sex, performance status, body mass index, and stage, with estimates standardized per 1-standard-deviation (SD) increase. **Results**: L3 SMI was associated with severe oral intake impairment (adjusted odds ratio per SD, 0.248; 95% CI, 0.053–0.891) and the treatment-to-discharge interval (adjusted β, −6.23 days; 95% CI, −11.89 to −0.58); the C3 index was not associated with any outcome. The standardized coefficients differed for oral intake impairment (*p* = 0.014) but not for other outcomes. Each binary outcome comprised only 10 events. **Conclusions**: L3 SMI was associated with nutritional vulnerability, whereas the fully automated C3 index was not; in an exploratory direct comparison, the two indices differed for severe oral intake impairment. These findings are hypothesis-generating and require confirmation in larger, multicenter cohorts.

## Simple Summary

Patients with head and neck cancer receiving chemoradiotherapy differ greatly in how well they tolerate treatment. Some develop severe swallowing problems, require a pause in radiation treatment, or take longer to be discharged, whereas others with similar disease complete treatment uneventfully. Thus, identifying those at highest risk early is necessary for planning supportive care. Loss of skeletal muscle has been proposed as a marker of reduced physical reserve, but it is unclear whether muscle measurements from the neck can reliably replace the standard measurement from the abdomen. In this study, we compared these two approaches using routine computed tomography scans obtained before treatment. We found that the abdominal muscle measurement, but not the neck measurement, was associated with severe swallowing impairment and a longer time from the start of treatment to discharge. These results suggest that the standard abdominal assessment may provide more useful information for identifying high-risk patients and may help guide earlier care planning.

## 1. Introduction

Patients with head and neck squamous cell carcinoma (HNSCC) receiving definitive chemoradiotherapy (CRT) exhibit substantial variability in treatment tolerance that is not fully explained by tumor stage, performance status, or body mass index (BMI). Some develop severe dysphagia requiring enteral feeding and remain hospitalized well beyond the scheduled treatment period, whereas others complete treatment without major complications despite similar baseline characteristics. This heterogeneity suggests that conventional clinical measures incompletely capture physiological reserve, and has motivated a growing interest in body composition as a pretreatment risk marker.

Loss of skeletal muscle mass has emerged as one of the most consistently reported markers in HNSCC [1,2]. Quantified on a single cross-sectional computed tomography (CT) image at the level of the third lumbar vertebra (L3) and normalized to height as the skeletal muscle index (SMI), low pre-treatment muscle mass has been associated with inferior overall and disease-free survival across multiple HNSCC cohorts [2,3]. Importantly, its clinical relevance extends beyond oncologic outcomes to treatment tolerance. Patients with low skeletal muscle mass are more likely to experience chemotherapy dose-limiting toxicity [4], require unplanned radiation treatment interruptions [5], and fail to complete concurrent CRT as planned [6]. These complications can compromise treatment delivery, delay discharge, and increase the need for supportive care.

However, there is a barrier to the routine clinical use of L3 SMI in this population. Abdominal imaging extending to L3 is not part of the standard diagnostic work-up for many patients with HNSCC, whose staging imaging is often confined to the head and neck [7]. To circumvent this, Swartz et al. proposed measuring the skeletal muscle at the level of the third cervical vertebra (C3), reliably captured on every head and neck scan, and developed a prediction equation to estimate L3 muscle mass from C3 measurements [7]. A subsequent study reported that C3-derived sarcopenia thresholds were independently associated with survival, supporting the use of the C3 approach as a convenient surrogate [8]. Because head and neck CT scans routinely include the cervical musculature, C3-based assessment offers a practical alternative when abdominal imaging is unavailable.

Yet the assumption that C3 faithfully reflects systemic muscle status has been challenged. In a study specifically examining patients with sarcopenia, the correlation between C3 and L3 muscle mass, which was strong in those without sarcopenia, deteriorated markedly among those with sarcopenia, with poor diagnostic agreement, particularly in patients for whom accurate classification is most clinically important [9]. These findings raise concerns about whether C3 can reliably substitute for L3 in all clinical settings—a question that, to our knowledge, has rarely been examined in relation to acute treatment-related complications. It therefore remains important to determine whether locoregional muscle (C3) provides information comparable to systemic muscle reserve (L3) when predicting the immediate, treatment-defining complications of CRT—severe oral intake impairment, radiation interruption, and a longer treatment-to-discharge interval—or whether anatomical convenience comes at the cost of predictive validity.

Recent advances in automated image analysis have also made the comparison increasingly feasible. Manual CT segmentation—once a major barrier to routine body composition analysis—has been substantially automated by deep learning tools such as TotalSegmentator, which segments anatomical structures across CT images with a performance approaching expert annotation [10,11]. Automated body composition pipelines built on such tools have been externally validated against manual measurements [12], raising the realistic prospect that muscle indices can be extracted from routine pretreatment imaging at minimal marginal cost, with no additional radiation, patient burden, or specialist time. Therefore, if a single body composition metric could be identified as the most informative for treatment-related outcomes, the path to clinical implementation would be unusually short.

Accordingly, we designed this exploratory study to address the question we believe is most relevant to multidisciplinary care: Among the body composition metrics now derivable automatically from pretreatment CT, does the conventional L3 SMI or the head-and-neck-specific C3 muscle index better identify, before treatment, the patients at risk of severe oral intake impairment and a longer treatment-to-discharge interval during definitive high-dose cisplatin-based CRT? Rather than anchoring our analysis to survival measured years downstream, we selected in-treatment events that drive supportive care decisions and inpatient resource use in a cohort treated with a uniform CRT regimen to minimize regimen heterogeneity that has complicated prior reports.

## 2. Materials and Methods

### 2.1. Study Design and Cohort

This single-center, retrospective, exploratory observational study conducted at a university hospital included patients enrolled between January 2023 and December 2024. The study protocol was approved by the institutional review board of our university hospital (approval number: HS2025-019), and information regarding the study was disclosed on the institutional website with an opt-out opportunity.

The inclusion criteria were as follows: (1) histologically confirmed SCC of the head and neck; (2) treatment with CRT at our institution during the study period; and (3) availability of pretreatment CT with body composition segmentation outputs. Exclusion criteria were as follows: (1) nasopharyngeal carcinoma, (2) SCC of unknown primary origin, and (3) unavailability of an L3 segmentation mask.

All patients were treated with definitive concurrent CRT on an inpatient basis. Radiotherapy was delivered using intensity-modulated radiotherapy (IMRT) at 2 Gy per fraction once daily for a total dose of 66 Gy in 33 fractions over approximately 7 weeks. The gross tumor volume (GTV) encompassed the primary tumor and involved lymph nodes. The clinical target volume (CTV) included the GTV with an appropriate margin and elective nodal regions at risk. Concurrent CRT consisted of cisplatin administered at 80 mg/m^2^ on days 1, 22, and 43 (three-weekly schedule, up to three cycles). Dose modifications or omissions of cisplatin were made at the discretion of the treating physician, based on organ function and tolerance.

### 2.2. Clinical Variables

Clinical data were extracted from the institutional electronic medical records and included age, sex, Eastern Cooperative Oncology Group (ECOG) performance status, height, body weight at admission and after completion of the third chemotherapy cycle, primary tumor site, clinical TNM classification (UICC 8th edition), clinical stage, serum albumin level, oral intake status at the conclusion of treatment, the most severe oral mucositis grade during treatment (the National Cancer Institute Common Terminology Criteria for Adverse Events version 5.0 [CTCAE v5.0]), occurrence of radiation interruption during the treatment course, and dates of treatment initiation and discharge.

### 2.3. Image Analysis

Pretreatment non-contrast CT images used for positron emission tomography (PET) fusion and attenuation correction were acquired using PET/CT systems from Siemens Healthineers (Erlangen, Germany: Biograph64 mCT, Biograph16 Horizon, and Biograph64 Vision), GE HealthCare (Chicago, IL, USA: Discovery STE and Discovery IQ), or Canon Medical Systems (Otawara, Japan: Celesteion). Acquisition parameters varied across scanners: slice thickness was 2.0–6.0 mm, tube voltage was 100–130 kV, and in-plane pixel spacing was 0.455–1.367 mm. The median volume computed tomography dose index (CTDI_vol_) was 4.09 mGy (range, 1.51–15.69 mGy). The images were reconstructed using scanner-specific soft-tissue kernels. All patients were scanned in the head-first supine position. Axial slices at the L3 and C3 vertebral levels were selected for body composition analysis. The CT images were segmented using TotalSegmentator (version 2.13.0) [10,13] implemented in 3D Slicer (version 5.10.0; www.slicer.org) [11]. Segmented structures, including the L3 and C3 vertebrae, skeletal muscle, visceral adipose tissue, subcutaneous adipose tissue, intermuscular adipose tissue, and head-and-neck muscle groups, were visually inspected for quality assurance.

**Figure 1:**
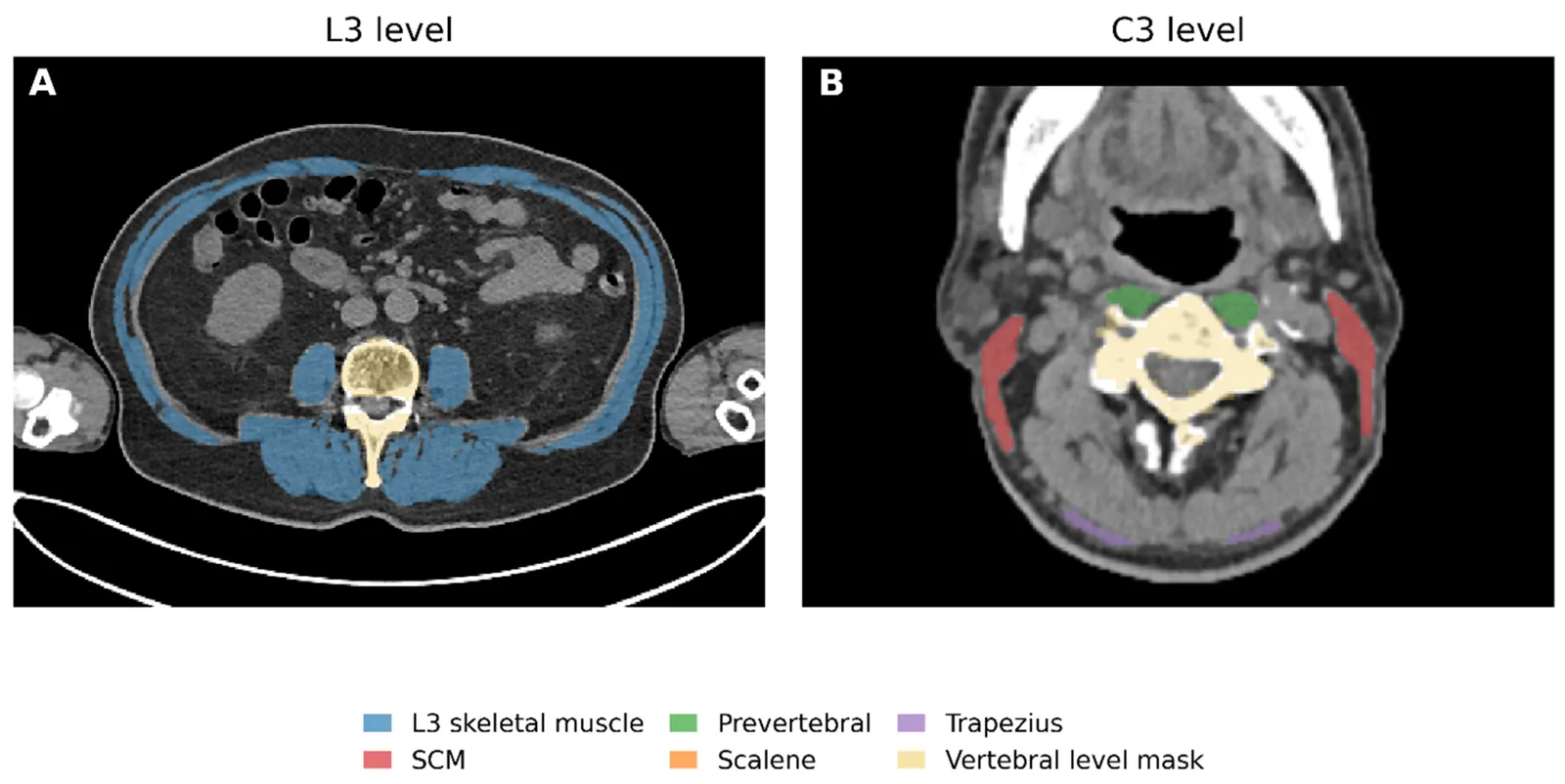
Representative examples of CT segmentation. Axial CT images from one representative case showing the segmented regions used for body composition measurements. (**A**) At the L3 level, the skeletal muscle within the torso was segmented to calculate the L3 skeletal muscle index. (**B**) At the C3 level, the sternocleidomastoid, prevertebral, scalene, and trapezius muscles were segmented to calculate the C3 head-and-neck muscle index. The vertebral level mask is indicated in yellow.

At the L3 level, the skeletal muscle cross-sectional area was used to calculate the L3 SMI. At the C3 level, the cross-sectional areas of the automatically segmented head-and-neck muscles, including the sternocleidomastoid, prevertebral, scalene, and trapezius muscles, were summed and normalized to height squared to calculate the C3 head-and-neck muscle index. Rather than reproducing previously published C3 protocols, we intentionally adopted the muscle groups directly generated by the automated segmentation pipeline to evaluate a fully automated workflow with minimal manual intervention, reflecting the intended clinical implementation of opportunistic body composition assessment. A representative example of the segmented regions used for these measurements is shown in Figure 1.

### 2.4. Outcomes

The primary binary outcome was severe oral intake impairment, defined as CTCAE v5.0 dysphagia grade ≥ 3, requiring parenteral nutrition, enteral tube feeding, or gastrostomy tube placement, with inability to maintain sufficient oral intake.

Secondary outcomes were treatment-to-discharge interval, the most severe oral mucositis grade, radiation interruption, pretreatment-to-post-treatment body weight loss (kg), and percentage body weight loss. The treatment-to-discharge interval was calculated as the number of days from the first day of radiotherapy to hospital discharge. Oral mucositis was graded according to CTCAE v5.0, using the ‘Mucositis oral’ item. Radiation interruption was defined as any unplanned break of ≥5 consecutive days (i.e., one treatment week) during the course of radiotherapy. Body weight loss was defined as body weight at admission minus body weight measured after the completion of the third cisplatin cycle, which approximately coincided with the end of radiotherapy. Percentage body weight loss was calculated as [(admission body weight − body weight after the third cisplatin cycle)/admission body weight] × 100. Discharge weight was not used to avoid confounding by the variable treatment-to-discharge interval, which was an outcome of interest.

### 2.5. Covariate Selection and Statistical Rationale

The pre-specified candidate confounders in the primary multivariable analyses were age, sex, ECOG performance status, BMI, and clinical stage. This reduced covariate set (five adjustment factors plus the primary predictor, totaling six predictors) was selected based on the following clinical and statistical rationale:

In patients with head and neck cancer undergoing radiotherapy or chemoradiotherapy, age, performance status, nutritional status including BMI or weight loss, and tumor stage have repeatedly been reported as clinically relevant predictors of enteral nutrition requirement and treatment-related vulnerability [14,15,16]. Treatment interruption is also clinically important in this population and may adversely affect oncological outcomes [17]. Sex was retained as a standard demographic covariate because body composition metrics are sex-dependent. Together, these five covariates were selected as a clinically parsimonious adjustment set to balance reproducibility, face validity, and model stability [18]. Clinical stage was entered as a single ordinal covariate scored 1, 2, 3, and 4 for stages I, II, III, and IV, respectively; stages IVA and IVB were both coded as 4. Thus, stage contributed one degree of freedom, with the coefficient representing an assumed linear change per one-stage increase.

The T and N classifications were considered but excluded. Simultaneously including stage, T classification, and N classification would constitute a triple adjustment for tumor burden in a dataset with only 10 events, leading to over-parameterization. Although large-scale analyses (e.g., Radiation Therapy Oncology Group trials) have used T and N as separate terms, the events-per-variable (EPV) ratio in this cohort (10 events for radiation interruption and 10 events for severe oral intake impairment among 55 complete cases) precluded the inclusion of more than six predictors without an unacceptable risk of overfitting.

Baseline albumin was retained as a descriptive baseline characteristic but excluded from the primary multivariable models to preserve a parsimonious covariate set; adding albumin would have increased the number of predictors and reduced the EPV ratio, and albumin overlaps conceptually with the nutritional dimension already captured by skeletal muscle mass. The prognostic nutritional index was excluded from modeling and tabulation because it is collinear with albumin and data quality concerns were identified on review. Cisplatin dose intensity (completed cycles or cumulative dose) was excluded from the models because it is determined during treatment and influenced by the same toxicities that define the study outcomes. As a post-baseline intermediate variable rather than a baseline confounder, its inclusion would risk over-adjustment and collider bias, attenuating the estimated effect of baseline body composition. Cisplatin delivery was reported descriptively but was not used as a covariate.

This parsimonious six-predictor set yielded an EPV ratio of approximately 1.7 for the binary outcomes (10 events across six predictors). Although restricting the covariate set reduced model complexity, the EPV remained substantially below conventional recommendations. Therefore, the multivariable analyses were considered exploratory and hypothesis-generating, and the adjusted estimates should be interpreted cautiously because residual overfitting and model instability cannot be excluded.

### 2.6. Statistical Analysis

Continuous variables are summarized as medians with interquartile ranges (IQRs) or as means with standard deviations, as appropriate. Categorical variables are summarized as n/N (%), with denominators shown in each table; missing categorical values are reported when relevant. Exploratory univariable analyses were performed using the Brunner–Munzel test for two-group comparisons and Spearman’s rank correlation for associations with continuous outcomes.

For multivariable analyses, we used a reduced exploratory model including six predictors: the primary body composition index, age, sex, ECOG performance status, BMI, and clinical stage. For binary outcomes (severe oral intake impairment and radiation interruption), Firth penalized logistic regression [19] was used as the primary estimator. This method applies a Jeffreys prior penalty to the likelihood, which reduces small-sample bias and yields finite estimates in the presence of quasi-complete separation—both common challenges when the EPV ratio is low. It was selected to improve estimation stability rather than to overcome the limited information available from a small number of events; it does not eliminate overfitting, guarantee precise estimates, or compensate for an inadequate sample size. Profile-likelihood 95% confidence intervals (CIs) were therefore reported to reflect estimation uncertainty, and the results were interpreted as exploratory associations rather than as a validated prediction model. Standard maximum likelihood logistic regression was additionally fitted as a sensitivity analysis to allow for comparisons with conventional approaches. For continuous outcomes, multivariable linear regression was fitted using the same reduced covariate set; the Firth penalty is specific to logistic regression and was therefore not applicable to these models. For the association between L3 SMI and the treatment-to-discharge interval, three sensitivity analyses were performed. First, standard errors for the same multivariable linear model were recomputed using the HC3 heteroskedasticity-consistent estimator. Second, the model was repeated after excluding each patient in turn to assess whether the estimate was driven by a single influential observation. Third, median regression was performed using the same covariates to examine the association with the conditional median rather than the conditional mean of the treatment-to-discharge interval. The L3 (primary predictor: L3 SMI) and head-and-neck models (primary predictor: C3 head-and-neck muscle index) were evaluated separately. All six model variables were complete for every patient in the analytic cohort, and the complete-case analyses therefore included all 55 patients without exclusion for missing data.

Because the two muscle indices differ substantially in scale—the standard deviation of L3 SMI (7.84 cm^2^/m^2^) is approximately 7.4 times that of the C3 head-and-neck muscle index (1.05 cm^2^/m^2^)—estimates expressed per 1 cm^2^/m^2^ are not directly comparable between them. To place the two indices on a common scale, each was standardized to a z-score and the models were refitted, yielding adjusted estimates per 1-standard-deviation increase. In addition, both indices were entered simultaneously into a single model for each outcome to assess whether either retained an association in the presence of the other. These models included seven predictors, corresponding to an EPV ratio of approximately 1.4 for the binary outcomes. Within these joint models, the null hypothesis that the two standardized coefficients were equal (H_0_: β_L3 = β_C3) was tested directly. For binary outcomes, the models were re-parameterized in terms of the sum and difference of the two standardized indices, and the difference term was tested by a profile penalized likelihood ratio test, with the ratio of the two odds ratios as the effect measure; for the continuous outcome, the corresponding linear contrast was tested by a Wald *t* test, with the difference in adjusted β (days per 1 SD) as the effect measure. Because these tests rest on 10 events per binary outcome and were performed for three outcomes without adjustment for multiplicity, they and the joint models above are reported as exploratory.

Statistical analyses were performed using Python 3.14 (statsmodels 0.14.6) and R 4.5.3 (logistf package 1.26.1). All tests were two-tailed, and a *p*-value less than 0.05 was considered statistically significant.

## 3. Results

### 3.1. Patients

**Figure 2:**
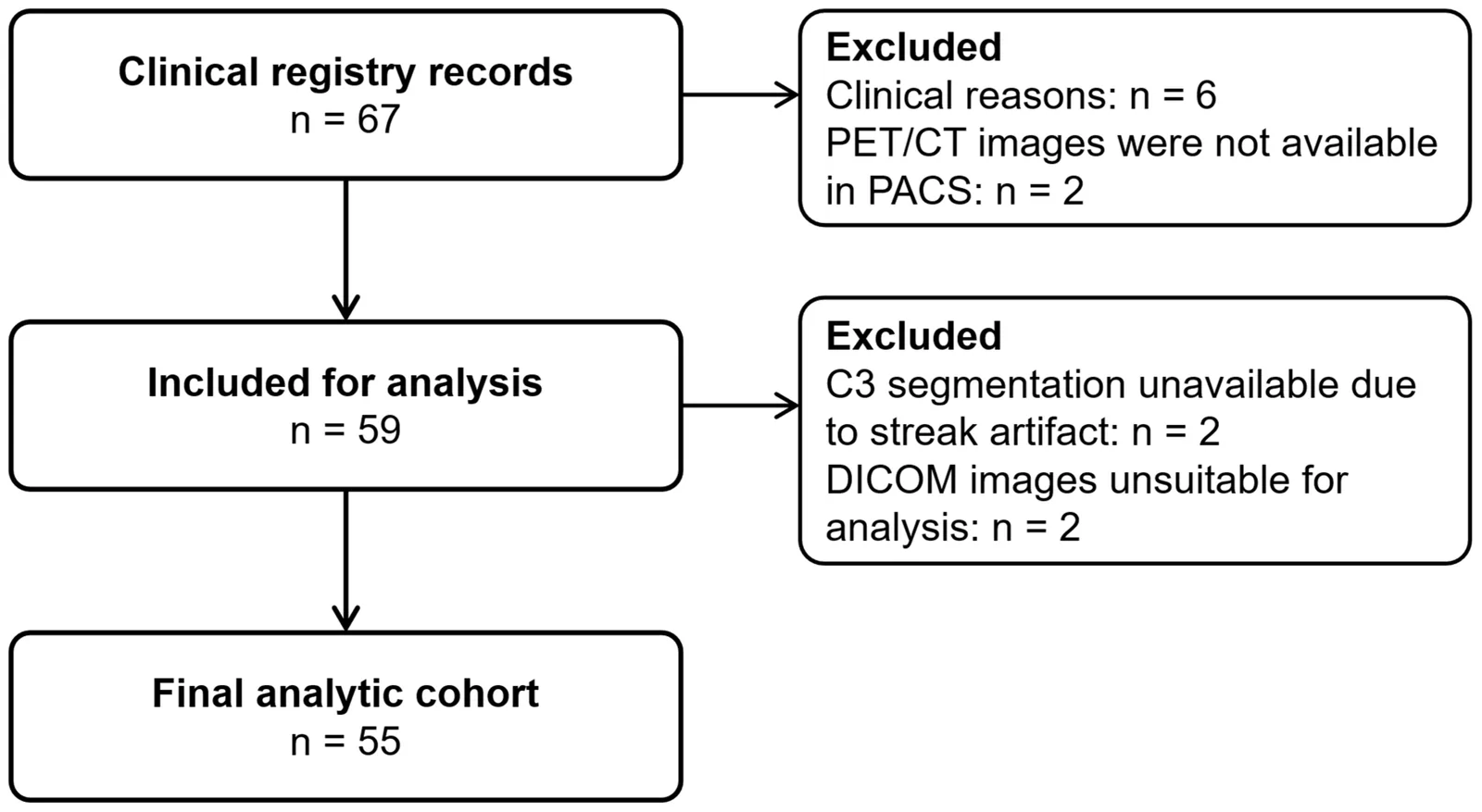
Flow diagram of the study cohort.

The analytical flow is illustrated in Figure 2. Among the 67 patients identified from the clinical registry, eight were excluded before image analysis: six for clinical reasons and two because PET/CT images were not available in the picture archiving and communication system. Thus, 59 patients proceeded to image analysis. Four additional patients were excluded because body composition assessment could not be completed: C3 segmentation was unavailable owing to streak artifacts in two patients, and Digital Imaging and Communications in Medicine images were unsuitable for analysis in two patients. The final cohort comprised 55 patients.

### 3.2. Patient Characteristics

The patient characteristics are summarized in Table 1. The most common primary cancer sites were the oropharynx (*n* = 28), hypopharynx (*n* = 19), and larynx (*n* = 7). The clinical stages were I–II in 21 patients, III in 20, and IV in 14. All included patients had M0 (no distant metastasis) disease. The median L3 SMI was 38.31 cm^2^/m^2^, and the median C3 head-and-neck muscle index was 3.35 cm^2^/m^2^. Regarding chemotherapy delivery, 51 of 55 patients (92.7%) completed all three planned cisplatin cycles, three (5.5%) completed two cycles, and one (1.8%) completed one or fewer cycles. The median cumulative cisplatin dose was 240 mg/m^2^ (IQR, 240–240).

### 3.3. Outcome Distributions

The distributions of the primary and secondary outcomes are shown in Table 2. Severe oral intake impairment and radiation interruption each occurred in 10 of 55 patients (18.2%). The most severe oral mucositis was grade 1 in 18 patients, grade 2 in 30 patients, and grade 3 in seven patients. The median treatment-to-discharge interval was 59.0 days, median body weight loss was 6.0 kg, and median percentage of body weight loss was 10.0%.

### 3.4. Exploratory Univariable Analyses

**Figure 3:**
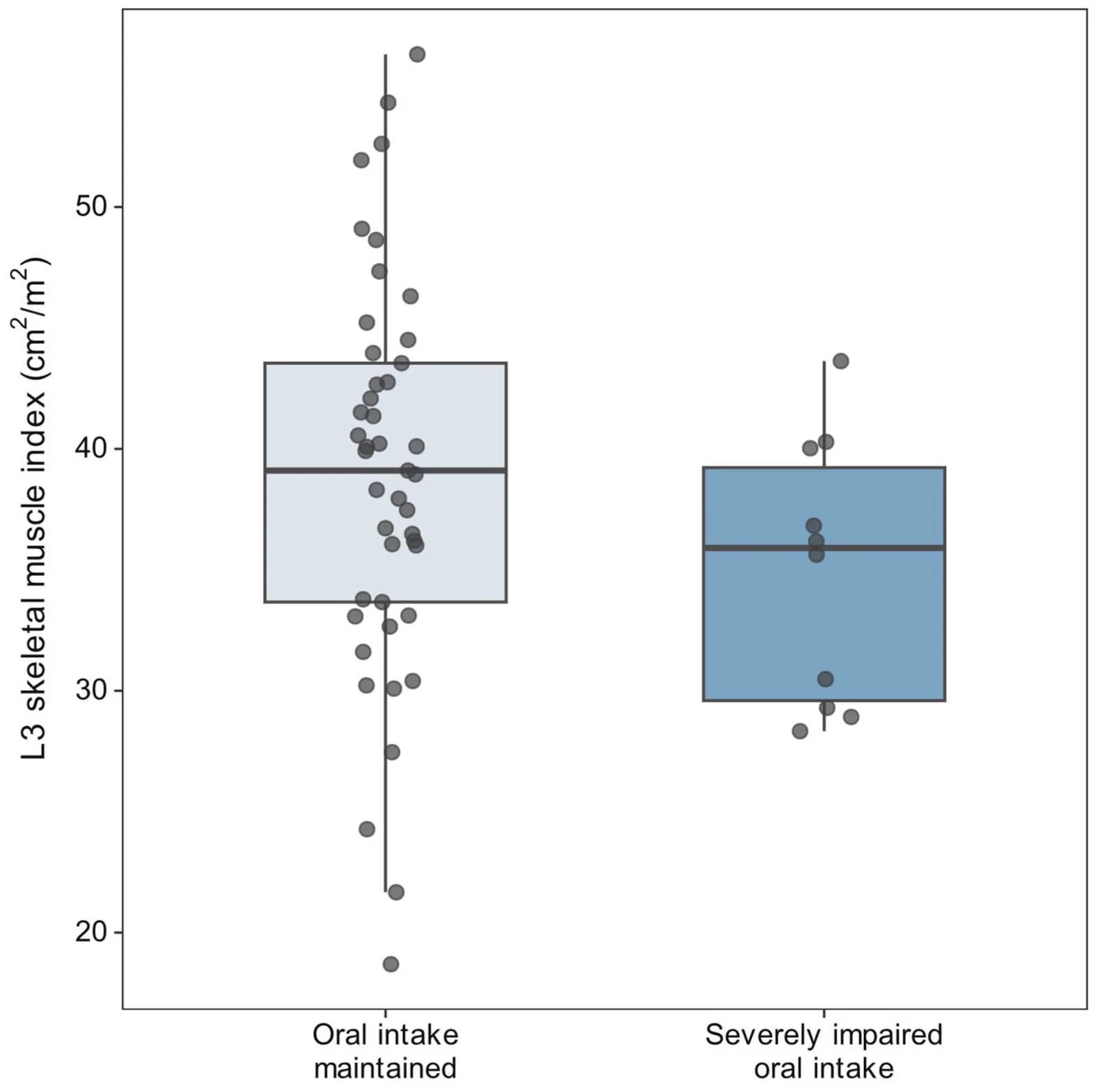
L3 SMI according to the feeding outcomes. Box plots of the L3 SMI stratified by feeding status. The severe oral intake impairment group had a lower median L3 SMI than the oral intake group, but the difference was not statistically significant (Brunner–Munzel test, *p* = 0.077). Boxes represent the interquartile range; horizontal lines within boxes show the median; whiskers extend to 1.5 × IQR; individual points indicate outliers.

**Figure 4:**
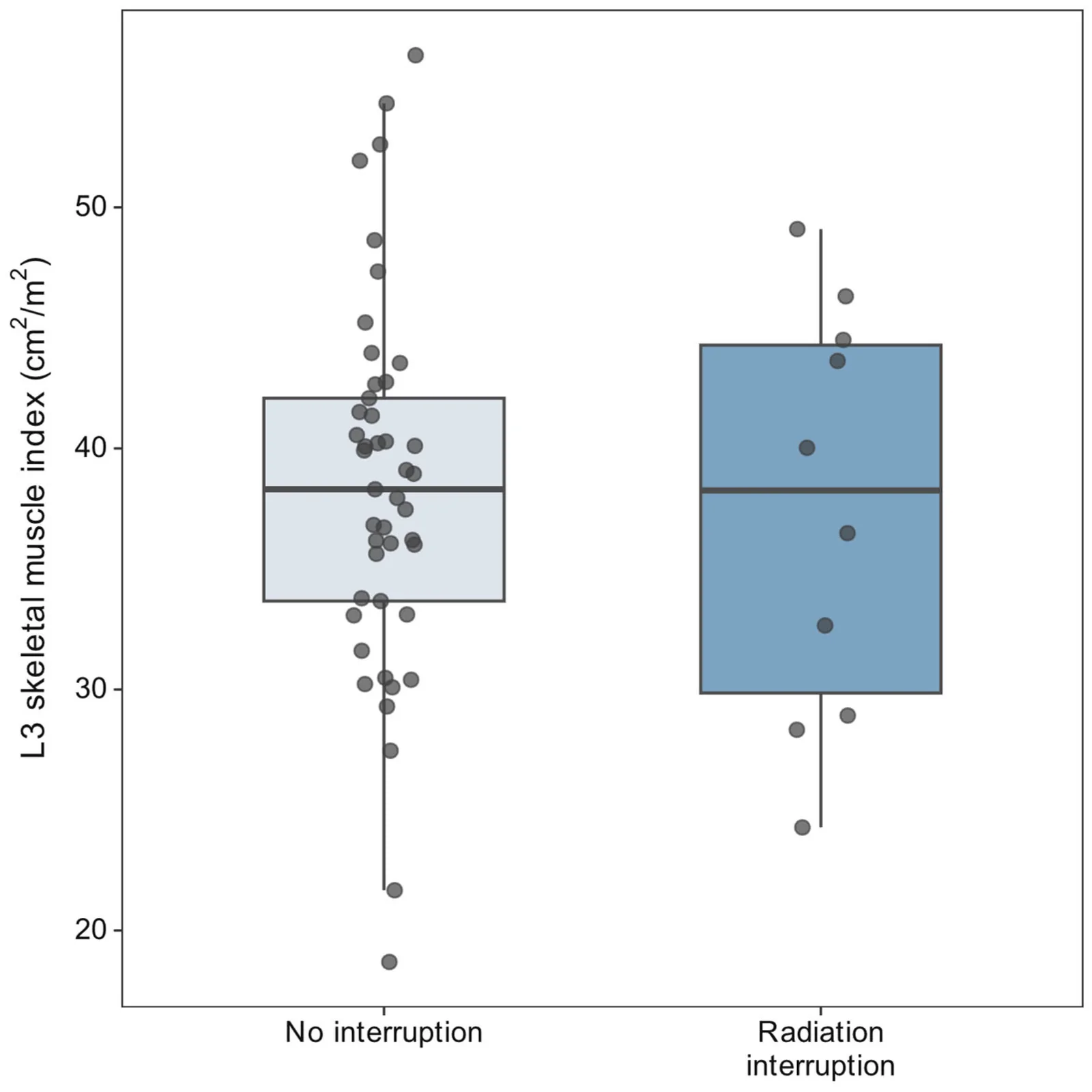
L3 SMI according to radiation interruption. Box plots of L3 SMI stratified by radiation interruption status (Brunner–Munzel test, *p* = 0.872). Boxes represent the interquartile range; horizontal lines within boxes show the median; whiskers extend to 1.5 × IQR; individual points indicate outliers.

The median L3 SMI was 35.9 cm^2^/m^2^ in the severe oral intake impairment group and 39.1 cm^2^/m^2^ in the oral intake maintained group, corresponding to a median difference of −3.2 cm^2^/m^2^ (severe impairment minus maintained; bootstrap 95% CI, −10.7 to 1.3). The Brunner–Munzel test did not provide sufficient evidence of between-group differences (*p* = 0.077; Figure 3). The median L3 SMI was 38.3 cm^2^/m^2^ in the radiation interruption group and 38.3 cm^2^/m^2^ in the no-interruption group, corresponding to a median difference of −0.05 cm^2^/m^2^ (radiation interruption minus no interruption; bootstrap 95% CI, −9.7 to 7.5). The Brunner–Munzel test did not provide evidence of between-group differences (*p* = 0.872; Figure 4).

**Figure 5:**
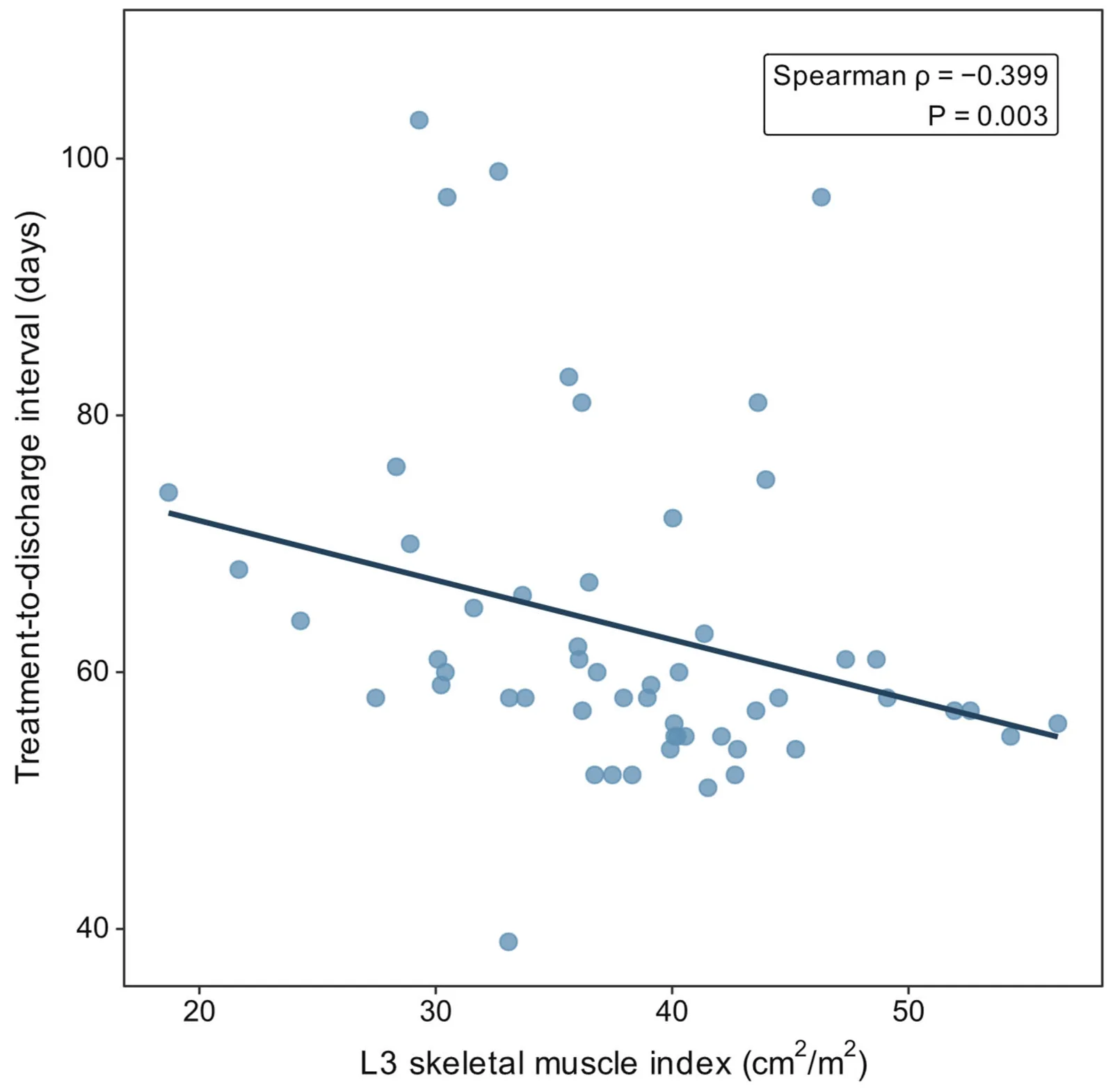
L3 SMI and treatment-to-discharge interval. Scatter plot with Spearman ρ = −0.399, *p* = 0.003. The solid line represents the linear least-squares regression fit, shown for visual reference only; the correlation coefficient was calculated using Spearman’s rank correlation.

Treatment-to-discharge interval was inversely correlated with L3 SMI (Spearman ρ = −0.399, *p* = 0.003; Figure 5). The most severe oral mucositis grade also showed an inverse association with L3 SMI (ρ = −0.253, *p* = 0.060). Body weight loss in kilograms was positively correlated with L3 SMI (Spearman ρ = 0.284, *p* = 0.036) and with the C3 head-and-neck muscle index (ρ = 0.391, *p* = 0.003) in univariable analyses.

### 3.5. Firth Penalized Logistic Regression for Binary Outcomes

The adjusted multivariable results for the binary outcomes are summarized in Table 3. In the L3 model, L3 SMI was not significantly associated with radiation interruption (adjusted odds ratio [OR], 0.968; 95% CI, 0.839–1.112; *p* = 0.640) but was inversely associated with severe oral intake impairment (adjusted OR, 0.837; 95% CI, 0.687–0.985; *p* = 0.032). In the head-and-neck model, the C3 head-and-neck muscle index was not significantly associated with radiation interruption (adjusted OR, 0.957; 95% CI, 0.471–2.158; *p* = 0.904) or severe oral intake impairment (adjusted OR, 1.533; 95% CI, 0.613–4.702; *p* = 0.400).

### 3.6. Multivariable Linear Regression for Continuous Outcomes

The adjusted multivariable linear regression models for the continuous outcomes are summarized in Table 4. In these models, L3 SMI was inversely associated with the treatment-to-discharge interval (adjusted beta, −0.795; 95% CI, −1.516 to −0.074; *p* = 0.032), whereas no clear association was observed with body weight loss in kilograms or with percentage body weight loss. The C3 head-and-neck muscle index showed no clear association with any continuous outcome. The inverse association between L3 SMI and the treatment-to-discharge interval remained when inference was based on HC3 heteroskedasticity-consistent standard errors (adjusted β, −0.795 days; 95% CI, −1.488 to −0.102; *p* = 0.025). In leave-one-out analyses, the coefficient remained negative throughout (range, −0.904 to −0.618 days), although the nominal *p* value exceeded 0.05 in 4 of 55 iterations, each following exclusion of a patient with a prolonged treatment-to-discharge interval. Median regression yielded a smaller negative estimate with a confidence interval that included the null value (adjusted β, −0.488 days; 95% CI, −1.131 to 0.154; *p* = 0.133). Thus, no single observation determined the direction or approximate magnitude of the association with the conditional mean, whereas the association with the conditional median remained uncertain.

### 3.7. Standardized Comparison of the L3 and C3 Muscle Indices

Because the standard deviations of the two indices differ by approximately 7.4-fold, adjusted estimates were also expressed per 1-standard-deviation increase to permit direct comparison (Table 5). On this common scale, L3 SMI remained associated with severe oral intake impairment (adjusted OR, 0.248; 95% CI, 0.053–0.891) and with the treatment-to-discharge interval (adjusted β, −6.23 days; 95% CI, −11.89 to −0.58), whereas the C3 index was associated with neither outcome (adjusted OR, 1.569; 95% CI, 0.597–5.114; and adjusted β, −0.80 days; 95% CI, −5.27 to +3.68, respectively). Neither index was associated with radiation interruption. Because standardization is a linear rescaling of the predictor, these *p* values are identical to those of the corresponding unstandardized models in Table 3 and Table 4.

In exploratory models containing both indices simultaneously, L3 SMI retained its association with severe oral intake impairment (adjusted OR, 0.197; 95% CI, 0.032–0.770) and with the treatment-to-discharge interval (adjusted β, −6.47 days; 95% CI, −12.45 to −0.50), whereas the C3 index was associated with no outcome (Table S1). Within these models, the null hypothesis that the two standardized coefficients were equal was tested directly. The coefficients differed for severe oral intake impairment (ratio of adjusted ORs, 0.074; 95% CI, 0.004–0.644; *p* = 0.014), but not for the treatment-to-discharge interval (difference in adjusted β, −7.10 days; 95% CI, −15.56 to +1.37; *p* = 0.098) or for radiation interruption (ratio of adjusted ORs, 0.762; 95% CI, 0.159–3.512; *p* = 0.720). These comparisons were derived from models with an EPV ratio of approximately 1.4 and were performed for three outcomes without adjustment for multiplicity.

### 3.8. Sensitivity Analysis Using Standard Logistic Regression

The results of the standard logistic regression sensitivity analysis were consistent with the Firth estimates. In the L3 model, the adjusted OR for severe oral intake impairment was 0.800 (95% CI, 0.652–0.983; *p* = 0.033), compared with 0.837 (95% CI, 0.687–0.985; *p* = 0.032) in the Firth model, and L3 SMI remained non-significant for radiation interruption (adjusted OR, 0.962; 95% CI, 0.826–1.121; *p* = 0.620). In the head-and-neck model, the C3 index remained non-significant for both outcomes. Full coefficient estimates from both estimators and both models are presented in Table S2.

## 4. Discussion

In this exploratory analysis of a uniformly treated cohort receiving high-dose cisplatin-based CRT, the pretreatment L3 SMI, but not the head-and-neck-specific C3 muscle index, was independently associated with severe oral intake impairment during treatment and the treatment-to-discharge interval. After adjustment for age, sex, performance status, BMI, and clinical stage, each one-standard-deviation decrease in L3 SMI corresponded to an approximately fourfold increase in the adjusted odds of severe oral intake impairment and a mean six-day longer treatment-to-discharge interval. Although these findings should be interpreted cautiously considering the exploratory design and limited sample size, the magnitude and direction of the association are clinically meaningful. Across the observed range of L3 SMI, this difference may translate into a clinically relevant variation in the probability of dysphagia developing during a curative treatment course.

The divergence between the univariable and adjusted analyses warrants comment. In the unadjusted comparison, L3 SMI did not differ significantly between patients with and without severe oral intake impairment (*p* = 0.077), whereas an association was evident after adjustment. Three factors plausibly contribute to this. First, the two analyses address different questions: the unadjusted test compares the marginal distribution of L3 SMI between outcome groups, whereas the regression estimates the association per unit of L3 SMI conditional on covariates. Second, adjustment for sex is material here, as skeletal muscle indices are strongly sex-dependent and 78% of the cohort was male; conditioning on sex removes between-sex variance that otherwise dilutes the marginal comparison. Third, a two-group comparison of a continuous exposure uses the available information less efficiently than a model that exploits its full range. Nonetheless, the absence of a significant unadjusted difference tempers confidence in the adjusted estimate, and we therefore regard this association as provisional and requiring confirmation in larger cohorts.

The dissociation between the L3 SMI and the C3 muscle index is, in our view, the most consequential finding of this study, and it speaks directly to an unresolved debate in the field. Following the introduction of the C3 prediction method by Swartz et al. [7], several groups reported that C3-derived muscle measurement was a convenient and prognostically valid surrogate for L3 [8]. This approach was rapidly adopted because head-and-neck imaging is universally available for this population, whereas abdominal imaging is not. Importantly, our study was not designed to reproduce previously published C3 protocols. Rather, we intentionally evaluated a fully automated workflow using the cervical muscle groups that can be directly segmented by the automated algorithm, without additional manual delineation or application of a C3-to-L3 prediction equation. This design was chosen to maximize feasibility for clinical implementation with minimal operator input and to assess the clinical utility of a fully automated opportunistic body composition workflow. Consequently, the resulting C3 head-and-neck muscle index differs from previously validated C3 methodologies, and our findings should be interpreted within that context. However, Yoon et al. demonstrated that the correlation between C3 and L3 muscle mass, which is robust in individuals without sarcopenia, deteriorates precisely in those with sarcopenia, the subgroup for whom accurate classification has the greatest clinical relevance [9]. Our findings extend this cautionary evidence beyond measurement agreement to clinical utility. In our cohort, the C3 index failed to reach the level of significance for any evaluated outcome, whereas L3 SMI was significantly associated with severe oral intake impairment and the treatment-to-discharge interval, but not with radiation interruption. Because statistical significance in one model and non-significance in another does not by itself establish that two indices differ, we tested the difference between their standardized coefficients directly within models containing both. The two indices differed for severe oral intake impairment (*p* = 0.014) but not for the treatment-to-discharge interval (*p* = 0.098) or radiation interruption (*p* = 0.720). Given 10 events per binary outcome, an events-per-variable ratio of approximately 1.4, and three comparisons without adjustment for multiplicity, this pattern is exploratory: it is consistent with, but does not establish, greater informativeness of L3 SMI for the nutritional endpoints in this setting. Conversely, with 10 events per binary outcome, the absence of a detectable association for the C3 index does not constitute evidence that it lacks utility, and our null results should not be read as excluding a clinically relevant association.

Neither muscle index was associated with radiation interruption, and this null result merits interpretation. The median L3 SMI was 38.3 cm^2^/m^2^ in both the interruption and no-interruption groups, with a median difference of −0.05 cm^2^/m^2^ (bootstrap 95% CI, −9.7 to 7.5), indicating that the point estimate lay essentially at the null rather than reflecting a true association obscured by imprecision. Two explanations appear plausible. First, in an inpatient setting with daily nursing and physician oversight, supportive measures are escalated promptly as toxicity develops. A patient with diminished muscle reserve may therefore be maintained on the planned schedule at the cost of intensified support, with that vulnerability manifesting instead as impaired oral intake and delayed discharge. Radiation interruption may accordingly be a less faithful marker of host physiological reserve than the nutritional endpoints in this care setting. Second, with only 10 interruption events, the analysis had limited power, and although the observed point estimate provides no indication of an association, a modest effect cannot be formally excluded.

Why might the systemic muscle reserve measured at a vertebral level remote from the irradiated field show a detectable association with treatment tolerance when muscle measured within or adjacent to the treatment volume did not? One possible explanation is that the two measurements reflect different aspects of physiological status. The L3 SMI may provide a more comprehensive marker of whole-body physiological reserve—the integrated capacity to withstand the cumulative metabolic burden of mucositis, dysgeusia, pain, nausea, and the systemic inflammatory milieu that characterizes the CRT course. Low skeletal muscle mass in cancer is increasingly understood not as an isolated anatomical finding but as a manifestation of a catabolic, pro-inflammatory state driven by tumor- and host-derived cytokines such as TNF-α and IL-6, which simultaneously degrade muscle and amplify treatment toxicity [20,21]. In contrast, the C3 measurement reflects a small, regionally specialized muscle group whose cross-sectional area may be a less reliable proxy for whole-body reserve. Even before any treatment is delivered, cervical soft tissues may be influenced by local factors, including the primary tumor, nodal disease, and peritumoral inflammation or edema that are unrelated to the systemic muscle status. Fundamentally, the limited and anatomically distinct musculature at C3 appears to diverge from the systemic muscle mass precisely in patients with sarcopenia [9], the subgroup in which the catabolic, pro-inflammatory state of advanced malignancy is most pronounced and most relevant to treatment tolerance. A patient may retain relatively preserved cervical musculature while harboring a depleted whole-body muscle reserve, which may not translate into better treatment tolerance. These mechanisms align with prior work showing that body composition predicts survival and locoregional control in head and neck cancer, independent of BMI and weight [22], reinforcing that it is the quality of the physiological reserve, not crude body size, that matters.

Our study extends existing literature in three clinically meaningful ways. First, the entire cohort received a uniform CRT regimen (cisplatin 80 mg/m^2^ every three weeks with 66 Gy IMRT), minimizing treatment heterogeneity that has complicated interpretation of previous reports combining diverse chemotherapy and radiation schedules [3,5]. Second, we directly compared the conventional L3 metric with the head-and-neck-specific C3 metric within the same patients, a head-to-head comparison that, despite its evident clinical relevance, has rarely been performed for in-treatment toxicity endpoints. Third, we focused on clinically actionable outcomes—not survival measured years downstream, but immediate events relevant to treatment completion and discharge, including severe oral intake impairment, unplanned radiation interruption, and a longer treatment-to-discharge interval. In this analysis, however, the evidence was strongest for severe oral intake impairment and treatment-to-discharge interval, whereas no significant association was observed with radiation interruption.

Should these associations be confirmed in larger cohorts, translating them into practice may require little additional infrastructure. First, where pretreatment imaging already includes the abdomen, L3 SMI can be derived at no additional cost, no additional radiation exposure, and no additional patient burden; and deep-learning segmentation tools, such as TotalSegmentator, now make automated measurement increasingly feasible [10,13]. Second, a low pretreatment L3 SMI may identify patients for whom anticipatory rather than reactive supportive care could be considered. Identifying high-risk patients before treatment would shift the clinical focus from detecting deterioration once it occurs to acting on a pretreatment risk signal. Concretely, such a pathway might comprise dietitian assessment before treatment initiation with individualized energy and protein targets, scheduled rather than symptom-triggered nutritional review during treatment, baseline swallowing assessment with prophylactic swallowing exercises, and a pre-treatment discussion of enteral access presented as contingency planning rather than as treatment failure. In the randomized experience of Paccagnella et al., early nutritional intervention in this population was associated with reduced weight loss, fewer treatment interruptions, and fewer unplanned admissions [23], and comparable principles underlie the emerging, if still maturing, literature on prehabilitation [24]. We would emphasize, however, that this evidence was not restricted to patients selected by muscle mass; whether muscle-mass-guided triage to intensified nutritional support improves outcomes over standard care is precisely the hypothesis our findings generate, and one that a prospective trial would be required to test. Third, the identification of such patients might warrant discussion of the chemotherapy schedule. Low skeletal muscle mass is an established predictor of cisplatin dose-limiting toxicity [4], and whether a low-dose weekly cisplatin schedule mitigates this risk in patients with muscle depletion is being examined in an ongoing randomized investigation [25]. We emphasize that these represent potential applications rather than recommendations; none has been prospectively evaluated in patients selected by muscle mass, and no validated threshold for action exists for the outcomes examined here.

Implementation is nonetheless not without friction. The principal constraint is anatomical coverage: L3 is captured only when staging or planning imaging extends to the abdomen, which is not routine at every center, and extending the scan field solely for body composition assessment would add radiation exposure and cost that the present evidence does not justify. Where such coverage already exists, the incremental burden is modest—segmentation is performed by open-source software without licensing cost and requires no additional patient contact—but visual quality assurance of each segmentation remains necessary, as two patients in our cohort could not be measured because of streak artifact at the C3 level. This step requires trained personnel and adds a small but non-zero radiology workload. Integration into clinical practice would also require a defined route by which the measurement reaches the multidisciplinary team before treatment begins, together with an agreed threshold for action; neither is currently established, since no validated cut-off exists for the outcomes examined here.

These findings should be weighed against the study’s limitations. First, this was a single-center study with a modest sample size, including 55 patients and only 10 events for each binary outcome. Although the covariate set was deliberately restricted to five clinically relevant adjustment factors plus the primary muscle index to limit model complexity [18], the resulting EPV ratio of approximately 1.7 remained low. To address sparse-data bias, Firth-penalized logistic regression was used because it provides more stable, finite estimates when conventional maximum likelihood estimation may be unstable [19]. However, Firth penalization cannot eliminate overfitting or compensate for the limited number of events. In addition, selection bias inherent to a retrospective single-center design cannot be excluded. Although all consecutive eligible patients were considered and the exclusions were determined solely by imaging availability and segmentation quality rather than by clinical course or outcome, the analytic cohort nevertheless reflects the referral pattern and treatment protocol of a single institution. The broadly consistent findings from the standard logistic regression sensitivity analysis suggest that the principal result was not solely dependent on the estimator, but do not establish model robustness. Therefore, these findings should be regarded as exploratory and require validation in larger, multicenter cohorts. We also note that the association between L3 SMI and severe oral intake impairment was not apparent in unadjusted analysis, which further underscores the provisional nature of these estimates. Second, the constraint of 10 events per binary outcome required a deliberately parsimonious covariate set, and three clinically relevant variables were consequently omitted. T and N classification was represented only indirectly through the composite UICC stage. Because stage collapses distinct patterns of tumor burden—a bulky T4N0 primary and a T1N3 nodal presentation may share a stage grouping yet differ substantially in irradiated mucosal volume and in the anatomical basis for dysphagia—residual confounding by tumor burden cannot be excluded, and the effect of local tumor extent on oral intake may be partly absorbed into the L3 SMI estimate. Baseline albumin was likewise omitted; since hypoalbuminemia correlates with both low muscle mass and treatment intolerance, its exclusion could bias the L3 SMI estimate away from the null. Our estimates should therefore be read as the association of low systemic muscle mass with adverse in-treatment outcomes in a model adjusted for stage but not for its components, rather than as an effect fully isolated from nutritional and tumor-burden confounding. Adequately powered cohorts permitting simultaneous adjustment for T classification, N classification, and albumin are required. For the same reason, interaction terms were not evaluated. Detecting effect modification requires substantially more events than estimating main effects, and any interaction estimated from 10 events would be too imprecise to interpret. Whether the association between L3 SMI and treatment tolerance is modified by age, performance status, or sex therefore remains an open question for adequately powered multicenter cohorts. Third, the case mix limits generalizability in two respects. Oropharyngeal carcinoma comprised 50.9% of the cohort, and 20 of these 28 patients (71.4%) were p16-positive; p16-positive disease therefore accounted for 20 of 55 patients (36.4%) overall, not for the cohort as a whole. Nevertheless, the oropharyngeal subgroup was p16-predominant, and because HPV-driven and HPV-negative disease differ in acute toxicity profile, nutritional trajectory, and prognosis, our estimates may not transfer to cohorts dominated by HPV-negative disease. Equally, with only one oral cavity case and a modest number of laryngeal cases, we cannot determine whether the L3 SMI association is uniform across primary sites, and site-specific dysphagia mechanisms make heterogeneity plausible. External validation in cohorts with differing HPV prevalence and site distribution is required. Fourth, we did not adjust for cisplatin dose intensity, as it is a post-baseline mediator rather than a baseline confounder of the causal pathway we explored. Fifth, the direct comparison of the two standardized muscle indices was performed in models containing seven predictors, corresponding to an events-per-variable ratio of approximately 1.4, and three outcomes were compared without adjustment for multiplicity. That comparison is therefore exploratory, and the difference observed for severe oral intake impairment requires confirmation before either index is preferred in practice. Finally, the C3 head-and-neck muscle index evaluated in this study was designed for a fully automated body composition workflow and therefore differs from several previously validated C3 methodologies, including protocols based on manually selected cervical muscles or C3-to-L3 prediction equations. Consequently, our findings should not be interpreted as applying to all C3-based assessment methods, and we cannot exclude the possibility that alternative validated C3 protocols would yield different results. Future studies directly comparing multiple C3 methodologies within the same cohort are warranted.

## 5. Conclusions

In this uniformly treated exploratory cohort, lower pretreatment L3 SMI was independently associated with severe oral intake impairment and a longer treatment-to-discharge interval, with each one-standard-deviation decrease corresponding to an approximately fourfold increase in the odds of severe oral intake impairment and a mean six-day longer interval to discharge. The fully automated C3 head-and-neck muscle index was not associated with any outcome and did not retain an association when both indices were modeled together; neither index was associated with radiation interruption. In an exploratory direct comparison of the standardized coefficients, the two indices differed for severe oral intake impairment but not for the treatment-to-discharge interval or radiation interruption; with 10 events per binary outcome, this comparison is hypothesis-generating and does not establish the superiority of either index. These findings suggest that systemic muscle reserve, obtainable from routine pretreatment imaging at negligible additional cost, may be the more informative body composition measure for anticipating nutritional vulnerability during chemoradiotherapy, but they remain hypothesis-generating and require confirmation in larger, multicenter cohorts before informing clinical triage.

## Figures and Tables

**Table 1:** Baseline patient characteristics.

Characteristic	Value
Number of patients	55
Age (years)	67.0 [57.5–71.0]
Sex: male	43/55 (78.2%)
Sex: female	12/55 (21.8%)
BMI (kg/m^2^)	21.31 [19.40–23.78]
Smoking status	
Ever	44/55 (80.0%)
Never	11/55 (20.0%)
Alcohol use	
None	15/55 (27.3%)
Current	40/55 (72.7%)
ECOG performance status 0	20/55 (36.4%)
ECOG performance status ≥ 1	35/55 (63.6%)
Primary site	
Oropharynx *	28/55 (50.9%)
p16-positive	20/28 (71.4%)
p16-negative	8/28 (28.6%)
Hypopharynx	19/55 (34.5%)
Larynx	7/55 (12.7%)
Oral cavity	1/55 (1.8%)
T classification	
T1	4/55 (7.3%)
T2	23/55 (41.8%)
T3	11/55 (20.0%)
T4	17/55 (30.9%)
N classification	
N0	19/55 (34.5%)
N1	18/55 (32.7%)
N2	11/55 (20.0%)
N3	7/55 (12.7%)
Clinical stage	
I–II	21/55 (38.2%)
III	20/55 (36.4%)
IV	14/55 (25.5%)
Albumin (g/dL)	4.20 [3.92–4.40]
L3 SMI (cm^2^/m^2^)	38.31 [33.09–42.71]
C3 head-and-neck muscle index (cm^2^/m^2^)	3.35 [2.79–4.01]
Chemotherapy delivery	
Completed all three planned cisplatin cycles	51/55 (92.7%)
Completed two cisplatin cycles	3/55 (5.5%)
Completed one cisplatin cycle or fewer	1/55 (1.8%)
Cumulative cisplatin dose delivered (mg/m^2^)	240 (240–240)

* Oropharyngeal carcinomas were staged according to p16 status in accordance with the UICC 8th edition. All categorical variables are presented using the full analytic cohort denominator (*n* = 55). Continuous variables are shown as medians [IQR]; baseline albumin was available for 45 of 55 patients. Abbreviations: BMI, body mass index; C3, third cervical vertebra; ECOG, Eastern Cooperative Oncology Group; IQR, interquartile range; L3, third lumbar vertebra; SMI, skeletal muscle index.

**Table 2:** Distribution of primary and secondary outcomes in the analytic cohort (*n* = 55).

Outcome	Value
Severe oral intake impairment	10/55 (18.2%)
Radiation interruption	10/55 (18.2%)
Most severe oral mucositis	
Grade 1	18/55 (32.7%)
Grade 2	30/55 (54.5%)
Grade 3	7/55 (12.7%)
Treatment-to-discharge interval (days)	59.0 [55.5–66.5]
Body weight loss (kg)	6.00 [4.00–8.40]
Body weight loss (%)	10.00 [6.54–14.46]

**Table 3:** Firth-penalized logistic regression analyses of binary outcomes.

Model	Outcome	Main Predictor	Complete-Case *n*	Events	Adjusted OR (95% CI)	*p* Value
L3 model	Radiation interruption	L3 SMI (cm^2^/m^2^)	55	10	0.968 (0.839 to 1.112)	0.640
	Severe oral intake impairment	L3 SMI (cm^2^/m^2^)	55	10	**0.837 (0.687 to 0.985)**	**0.032**
Head-and-neck model	Radiation interruption	C3 head-and-neck muscle index (cm^2^/m^2^)	55	10	0.957 (0.471 to 2.158)	0.904
	Severe oral intake impairment	C3 head-and-neck muscle index (cm^2^/m^2^)	55	10	1.533 (0.613 to 4.702)	0.400

Adjusted ORs are expressed per 1 cm^2^/m^2^ increase and were obtained by Firth-penalized logistic regression with profile-likelihood 95% CIs, adjusted for age, sex, ECOG performance status, BMI, and clinical stage. Bold indicates a 95% CI excluding 1.00. Abbreviations: C3, third cervical vertebra; CI, confidence interval; L3, third lumbar vertebra; OR, odds ratio; SMI, skeletal muscle index.

**Table 4:** Multivariable linear regression analyses of continuous outcomes.

Model	Outcome	Main Predictor	Complete-Case *n*	Adjusted R^2^	Adjusted Beta (95% CI)	*p* Value
L3 model	**Treatment-to-discharge interval (days)**	**L3 SMI (cm^2^/m^2^)**	**55**	**0.090**	**−0.795 (−1.516 to −0.074)**	**0.032**
	Body weight loss (kg)	L3 SMI (cm^2^/m^2^)	55	0.133	0.019 (−0.166 to 0.203)	0.840
	Body weight loss (%)	L3 SMI (cm^2^/m^2^)	55	−0.004	0.052 (−0.256 to 0.361)	0.734
Head-and-neck model	Treatment-to-discharge interval (days)	C3 head-and-neck muscle index (cm^2^/m^2^)	55	0.000	−0.756 (−4.998 to 3.486)	0.722
	Body weight loss (kg)	C3 head-and-neck muscle index (cm^2^/m^2^)	55	0.152	0.541 (−0.484 to 1.565)	0.294
	Body weight loss (%)	C3 head-and-neck muscle index (cm^2^/m^2^)	55	−0.003	0.376 (−1.351 to 2.103)	0.663

Adjusted beta coefficients were obtained by multivariable linear regression adjusted for age, sex, ECOG performance status, BMI, and clinical stage. Negative adjusted R^2^ values indicate no explanatory gain over chance. Abbreviations: C3, third cervical vertebra; CI, confidence interval; L3, third lumbar vertebra; R^2^, coefficient of determination; SMI, skeletal muscle index.

**Table 5:** Standardized (per 1-SD) adjusted associations of the L3 skeletal muscle index and the C3 head-and-neck muscle index with study outcomes.

Outcome	Index	Adjusted Estimate (95% CI), Per 1-SD Increase	*p* Value
***Binary outcomes—adjusted odds ratio***			
**Severe oral intake impairment**	**L3 SMI**	**0.248 (0.053 to 0.891)**	**0.032**
	C3 head-and-neck muscle index	1.569 (0.597 to 5.114)	0.400
Radiation interruption	L3 SMI	0.773 (0.252 to 2.293)	0.640
	C3 head-and-neck muscle index	0.954 (0.452 to 2.250)	0.904
***Continuous outcome—adjusted β (days)***			
**Treatment-to-discharge interval**	**L3 SMI**	**−6.23 (−11.89 to −0.58)**	**0.032**
	C3 head-and-neck muscle index	−0.80 (−5.27 to +3.68)	0.722

Estimates are expressed per 1-standard-deviation (SD) increase in each index (L3 SMI, 7.84 cm^2^/m^2^; C3 head-and-neck muscle index, 1.05 cm^2^/m^2^) and are adjusted for age, sex, ECOG performance status, BMI, and clinical stage (*n* = 55; 10 events for each binary outcome). Each index was entered in a separate model. Binary outcomes are reported as adjusted ORs obtained by Firth-penalized logistic regression with profile-likelihood 95% CIs; the continuous outcome as adjusted β coefficients (days) obtained by multivariable linear regression. Bold indicates a 95% CI excluding the null value (1.00 for ORs and 0 for β coefficients). Because standardization is a linear rescaling of the predictor, the *p* values are identical to those of the corresponding unstandardized models in Table 3 and Table 4. Estimates from models containing both indices simultaneously are provided in Table S1. Abbreviations: BMI, body mass index; C3, third cervical vertebra; CI, confidence interval; ECOG, Eastern Cooperative Oncology Group; L3, third lumbar vertebra; OR, odds ratio; SD, standard deviation; SMI, skeletal muscle index.

## Data Availability

Data presented in this study are available upon request from the corresponding author. The data are not publicly available due to privacy and ethical restrictions.

## Supplementary Materials

The following supporting information can be downloaded at: https://www.mdpi.com/article/10.3390/cancers18162677/s1, Table S1: Adjusted associations from models containing both the L3 skeletal muscle index and the C3 head-and-neck muscle index simultaneously, and direct comparison of their standardized coefficients (exploratory); Table S2: Full coefficient estimates from the Firth-penalized and standard logistic regression analyses of radiation interruption and severe oral intake impairment.
