# Supplementary material for: Pretreatment L3 Skeletal Muscle Index Is Associated with Severe Oral Intake Impairment and a Longer Treatment-to-Discharge Interval in Patients with Head and Neck Squamous Cell Carcinoma Receiving Definitive Chemoradiotherapy
Source: Cancers (Basel). 2026 Aug 18;18(16):2677. doi: 10.3390/cancers18162677 (PMC13511419; doi:10.3390/cancers18162677)
Supplement: Supplementary file 1 [file cancers-18-02677-s001.zip › cancers-4416204-supplementary.pdf]

cancers

MDPI

Supplementary Materials

Table S1. Adjusted associations from models containing both the L3 skeletal muscle index and the C3 head-and-neck muscle index simultaneously, and direct comparison of their standardized coefficients (exploratory).

Outcome	Index	Adjusted estimate (95% CI), per 1-SD increase	P value
Binary outcomes — adjusted odds ratio			
**Severe oral intake impairment**	**L3 SMI**	**0.197 (0.032 to 0.770)**	**0.017**
	C3 head-and-neck muscle index	2.662 (0.721 to 13.688)	0.157
Radiation interruption	L3 SMI	0.768 (0.249 to 2.361)	0.639
	C3 head-and-neck muscle index	1.008 (0.470 to 2.481)	0.985
Continuous outcome — adjusted β (days)			
**Treatment-to-discharge interval**	**L3 SMI**	**−6.47 (−12.45 to −0.50)**	**0.034**
	C3 head-and-neck muscle index	+0.62 (−3.88 to +5.13)	0.782
Direct comparison of the two indices — H_0: equal standardized coefficients			
**Severe oral intake impairment**	**L3 SMI vs C3 index (ratio of ORs)**	**0.074 (0.004 to 0.644)**	**0.014**
Radiation interruption	L3 SMI vs C3 index (ratio of ORs)	0.762 (0.159 to 3.512)	0.720
Treatment-to-discharge interval	L3 SMI vs C3 index (difference in β, days)	−7.10 (−15.56 to +1.37)	0.098

Binary outcomes are reported as adjusted odds ratios (ORs), and the continuous outcome as adjusted β coefficients. Both muscle indices were entered simultaneously into a single model for each outcome, together with age, sex, Eastern Cooperative Oncology Group (ECOG) performance status, body mass index (BMI), and clinical stage, yielding seven predictors per model (n = 55). Estimates are expressed per 1-standard-deviation (SD) increase in each muscle index. The two indices were moderately correlated (Pearson r = 0.60; variance inflation factor = 1.55).

Binary outcomes were analyzed using Firth-penalized logistic regression with profile-likelihood 95% confidence intervals (CIs), and the continuous outcome was analyzed using multivariable linear regression. Bold indicates a 95% CI excluding the null value (1.00 for ORs and 0 for β coefficients). Direct comparisons tested the null hypothesis that the standardized coefficients of the two muscle indices were equal within the same model. For binary outcomes, the model was reparameterized using the sum and difference of the two standardized indices, and the coefficient of the difference term was tested using a profile penalized likelihood-ratio test; the effect measure is the ratio of the ORs for L3 SMI relative to the C3 head-and-neck muscle index. For the continuous outcome, a Wald t test of the linear contrast was used, and the effect measure is the difference in adjusted β coefficients (days per 1-SD increase).

With only 10 events per binary outcome, the events-per-variable ratio was approximately 1.4. In addition, three outcomes were compared without adjustment for multiple comparisons. These analyses are therefore exploratory and should not be interpreted as confirmatory evidence.

Abbreviations: BMI, body mass index; C3, third cervical vertebra; CI, confidence interval; ECOG, Eastern Cooperative Oncology Group; L3, third lumbar vertebra; OR, odds ratio; SD, standard deviation; SMI, skeletal muscle index.

Table S2. Full coefficient estimates from the Firth-penalized and standard logistic regression analyses of radiation interruption and severe oral intake impairment.

Predictor	Firth penalized logistic regression, Adjusted OR (95% CI)	P value	Standard logistic regression, Adjusted OR (95% CI)	P value
**L3 Model — Radiation Interruption (n = 55, events = 10)**				
L3 SMI (cm²/m²)	0.968 (0.839 to 1.112)	0.64	0.962 (0.826 to 1.121)	0.62
Age (years)	1.001 (0.920 to 1.089)	0.987	1.003 (0.916 to 1.100)	0.941
Sex (female)	0.631 (0.073 to 4.544)	0.648	0.501 (0.053 to 4.764)	0.547
ECOG PS	1.027 (0.224 to 5.219)	0.973	1.053 (0.193 to 5.754)	0.953
BMI (kg/m²)	1.022 (0.731 to 1.380)	0.893	1.014 (0.718 to 1.430)	0.938
Clinical stage	0.803 (0.339 to 1.866)	0.607	0.761 (0.304 to 1.904)	0.559
**L3 Model — Severely Impaired Oral Intake (n = 55, events = 10)**				
**L3 SMI (cm²/m²)**	**0.837 (0.687 to 0.985)**	**0.032**	**0.800 (0.652 to 0.983)**	**0.033**
Age (years)	0.958 (0.855 to 1.063)	0.413	0.948 (0.839 to 1.072)	0.395
Sex (female)	0.151 (0.008 to 1.626)	0.121	0.075 (0.004 to 1.553)	0.094
**ECOG PS**	**0.154 (0.016 to 0.949)**	**0.044**	**0.091 (0.009 to 0.939)**	**0.044**
BMI (kg/m²)	1.107 (0.752 to 1.554)	0.568	1.114 (0.743 to 1.670)	0.602
**Clinical stage**	**3.655 (1.250 to 16.092)**	**0.016**	**5.310 (1.201 to 23.479)**	**0.028**
**Head-Neck Model — Radiation Interruption (n = 55, events = 10)**				
C3 head-neck muscle index (cm²/m²)	0.957 (0.471 to 2.158)	0.904	0.983 (0.428 to 2.260)	0.968
Age (years)	1.009 (0.937 to 1.091)	0.811	1.013 (0.934 to 1.100)	0.75
Sex (female)	0.753 (0.100 to 4.655)	0.764	0.638 (0.079 to 5.145)	0.673
ECOG PS	0.936 (0.210 to 4.536)	0.932	0.963 (0.184 to 5.038)	0.965
BMI (kg/m²)	0.985 (0.730 to 1.242)	0.905	0.959 (0.717 to 1.283)	0.779
Clinical stage	0.785 (0.332 to 1.826)	0.57	0.742 (0.297 to 1.849)	0.521
**Head-Neck Model — Severely Impaired Oral Intake (n = 55, events = 10)**				
C3 head-neck muscle index (cm²/m²)	1.533 (0.613 to 4.702)	0.4	1.835 (0.596 to 5.653)	0.29
Age (years)	1.010 (0.919 to 1.118)	0.84	1.018 (0.913 to 1.136)	0.745
Sex (female)	0.572 (0.043 to 4.997)	0.621	0.391 (0.026 to 5.825)	0.495
**ECOG PS**	**0.148 (0.020 to 0.780)**	**0.024**	**0.090 (0.011 to 0.719)**	**0.023**
BMI (kg/m²)	0.807 (0.489 to 1.129)	0.262	0.729 (0.453 to 1.174)	0.193
Clinical stage	2.405 (0.910 to 8.535)	0.079	3.044 (0.862 to 10.746)	0.084

Values are adjusted odds ratios (ORs) with 95% confidence intervals (CIs). Given only 10 events per binary outcome, each exploratory model included one primary muscle index and five prespecified adjustment variables: age, sex, Eastern Cooperative Oncology Group (ECOG) performance status, body mass index (BMI), and clinical stage. L3 SMI and the C3 head-and-neck muscle index were modeled separately. Firth-penalized logistic regression was used as the primary analysis to reduce small-sample bias and instability under sparse data, and standard maximum-likelihood logistic regression was performed as a sensitivity analysis. Male sex was the reference category. Clinical stage was entered as a single ordinal covariate (I = 1, II = 2, III = 3, and IV = 4; stages IVA and IVB were coded as 4). ECOG performance status and clinical stage were coded as described in the Methods. Bold indicates a 95% CI excluding 1.00.

Abbreviations: BMI, body mass index; C3, third cervical vertebra; CI, confidence interval; ECOG PS, Eastern Cooperative Oncology Group performance status; L3, third lumbar vertebra; OR, odds ratio; SMI, skeletal muscle index.
